# The Latest Experiments in Contagion and Prophylactic Inoculation Summed up

**Published:** 1880-11

**Authors:** H. D. Valin

**Affiliations:** 186 Blue Island Ave.; Chicago


ARTICLE III.

### THE LATEST EXPERIMENTS IN CONTAGION AND PROPHYLACTIC INOCULATION SUMMED UP. By H. D. VALIN, M.D., Chicago.

The author in his Inaugural Thesis on the Philosophy of Disease, presented to the medical faculty of the University of Vermont, in 1879, recapitulated thus the views held at that time by the best writers on the germ theory of disease: "The contagium of infectious diseases is regarded by some authors as too minute to be discovered by the microscope; by others, as bacteria which cannot be distinguished from those generally found in many pathological processes in the body. The spread of the contagium requires a region (an habitat) wherein it has always been endemic, as India for cholera, and Ireland for typhus fever, unless we accept for it a theory of spontaneous generation. It also requires a certain age, sex, and predisposition on the part of the individual, and its extension is promoted by warmth, moisture and filth; these accessory circumstances explaining how there may be degrees in contagion. Each infectious disease must, beside, have special bacteria assigned as its cause."

These conclusions, drawn from a perusal of the first three volumes of Ziemssen's Cyclopædia of the Practice of Medicine, were little more than theoretical, but recent experiments have strengthened the germ theory, and it may finally fill the gap so long left in the etiology of the most important diseases.

Prof. Lister read before the annual meeting of the British Medical Association an interesting paper upon "Micro-organisms:

their Relation to Disease." It is thus recapitulated in the *N. Y. Medical Record:*

"Prof. Lister first detailed the results of the experiments of Dr. Koch, of Wolstein. By ingenious methods of staining, that investigator had been able to detect not only the *bacillus anthracis*, which causes anthrax, but he discovered other specific micro-organisms. These were the *bacillus septicæmiæ* and the chain *micrococcus*, the latter always causing gangrene when injected into house mice.

"The *bacillus septicæmiæ* is an extremely minute organism, and would be invisible unless stained. Dr. Koch had been able to prove that it always caused septicæmia, never pyæmia, when injected into mice.

"Prof. Lister then described the micro-organism discovered by Toussaint in fowls suffering from fowl-cholera. This fowl-cholera is a highly infectious blood disease, characterized by the swelling of the lymphatics of the neck, pericarditis and duodenitis, but without diarrhœa. The bacterium claimed to be the cause of the fowl-cholera, is oval shaped, multiplies by transverse constriction, and is about $\frac{1}{40,000}$ of an inch in diameter. Pasteur has cultivated this organism, and has been able to modify it, so that by injecting the modified fluid he can cause a mild form of fowl-cholera—protective against a second attack. In the same line of inquiry, Dr. Greenfield, by experiments, has recently shown that if a guinea-pig be inoculated with the blood of a heifer suffering from splenic fever, the former animal takes the disease; if now the blood of the guinea-pig be inoculated in a healthy heifer, it will protect it against splenic fever. Prof. Toussaint had performed similar experiments in regard to 'vaccinating' for anthrax. But he went further; he showed that the protective power is not conferred *by inoculating any form of the bacillus anthracis, but by inoculating the fluid in which it had grown.*"*

In conclusion, the speaker describes the ingenious experiments of Dr. Buchner, of Munich, by which he had transformed, by cultivation, the *bacillus anthracis* into the hay-bacillus and *vice versa*. A record of some of those highly interesting experiments follows as translated from the originals.

* Italics ours.

Dr. Edouard Fournié, in his periodical,* thus analyzes the experiments on anthrax:

"The question of the contagious elements of disease has lately been revived, thanks to the communications of Pasteur, Colin (of Alfort), Chauveau, Poincaré and Toussaint on anthrax.

The paper which Pasteur lately presented to the Institute and the Academy of Medicine (Paris), aims at showing experimentally the nature and pathogeny of anthrax. The author does not recognize any spontaneous origin of diseases. Every one of these has its material cause, and anthrax is always produced by the *bacillus anthracis.* That was the point to be demonstrated.

In a first series of experiments, Pasteur fed several sheep some lucern which had been sprinkled with water holding bacteria and germinal corpuscles of the latter. In order to facilitate inoculation through abrasions, care was taken to mix thistles and beards of barley with the food. It resulted in the death of a few, and the survival of the majority of the animals, notwithstanding the enormous quantity of the bacteria injected. Autopsy showed the characteristic lesions of anthrax, having their starting point in the mouth and the pharynx.

In another series of experiments, Pasteur inoculated eight sheep with bacteria. All were taken sick with a rise in their temperature, but only one died; one had been inoculated under the tongue. These unexpected results contravene our knowledge the more as Pasteur claims to vaccinate efficiently fowls by submitting to them a food contaminated by the bacteria of fowl cholera.

The foregoing experiments led Pasteur to suspect that cattle which die suddenly with anthrax, in the department of Eure-et-Loir, are themselves infected with the *bacillus anthracis* mixed with their food. But where would the germinal corpuscles come from?

Pasteur discovered that their origin was in the cadavers of animals dead from anthrax and buried in the soil. Davaine and Colin had remarked that bacteria are destroyed by putrefaction, but their germinal corpuscles, according to Pasteur, resist that

* Revue Médicale Franç. et Etrangère, July 31st.

influence, and these on being carried to a favorable medium pass from a latent to an active life and become bacteria.

After digging some earth from the bottom of pits in which cattle dead from anthrax had been buried two years previously, Pasteur found germinal corpuscles in it, and succeeded in inoculating and killing animals with anthrax.

The manner in which anthrax germs come to the surface is explained thus: It is shown that earthworms, after feeding on the corrupted dirt come to the surface where they deposit their excrement which contains a large number of germinal corpuscles. These, after drying are blown by the wind in all directions, on plants, flowers and on the abrasions common on the skin of cattle.

Says Pasteur: "The results suggest the possible influence of soils in the etiology of disease, the danger which may result from graveyards, the usefulness of cremation!"

The experiments lately reported to the Institute, by Dr. Poincaré, substantiate the researches of Pasteur.

Nineteen cattle having died of anthrax, on a farm in the vicinity of Nancy, Mr. Tisserand, a veterinary surgeon, thought the cause of that local epizootic should be referred to a stream of muddy water flowing through the pasture. Dr. Poincaré demonstrated the presence of bacteria in the blood of the diseased animals, and in water from the stream; and he injected some of the water into an animal which soon died with characteristic symptoms of anthrax.

Some time ago, Dr. Chauveau communicated to the Institute the results of experiments which he made in Algeria, with an aim at determining the nature of the immunity of Algerian sheep from anthrax.

These were his conclusions:

"All the sheep indigenous to Algeria enjoy in a lesser or greater degree an immunity from splenic fever, and can impart this immunity to European sheep by crossing. That immunity is congenital and natural. French sheep, breeding in Algeria, do not acquire it, but it is not proven that Algerian sheep, breeding in France, may not lose it. Hence we are not allowed to deny some influence to the Algerian medium, at least in what pertains to the preservation of that immunity which Algerian sheep possess."

An eminent experimenter, Prof. Toussaint (of Toulouse), has just brought to the notice of the Institute facts of great interest. He has ascertained, as a first step, that bacteria, even in the animals in which anthrax is most apt to occur, do not reach their maturity in the body where they are first received and which they destroy; they never produce germinal corpuscles in it, but multiply through a division of the mycelium. Furthermore, he has ascertained that some animals, as the swine, never take anthrax, although their conditions of life resemble very much those of other species attacked most readily. He also noticed that some animals easily catch anthrax in their youth, and lose that predisposition in adult life and old age, e. g., the dog, the horse, the ass and others.

These facts led Toussaint to inquire into the conditions which render an organism proof against the action of bacteria. His manner of proceeding consists in depriving the blood of animals sick with anthrax of its bacteria through filtering, in raising its temperature to F. 130°, and inoculating it in the groin and the neck in the neighborhood of lymphatics. The result of the inoculation is slightly manifested locally, and the blood is modified and becomes proof against the growth of the *bacillus anthracis.*

The following are his conclusions:

1. The blood of animals suffering from anthrax, divested of its bacteria, does not communicate that disease, but an affection *sui generis* which imparts to the organism an immunity against anthrax.

2. The germinal corpuscles, whose office is so important according to Pasteur, do not exist any longer in the blood after filtering.

3. An infectious liquid may produce a pathological change, although deprived of bacteria and germinal corpuscles.

4. The lymphatics have a necessary office in the production of infection, as previously proven by Cohn.

Here is an extract and translation from another important paper presented to the French Institute by Dr. M. P. Miquel,*

*Revue Méd. Franç, et Etrang, July 24th.

stating some of the laws which govern the spread of the germinal corpuscles of bacteria:

"Through processes of breeding, the details of which are too full to state here, I have succeeded in securing and enumerating the germs of bacteria, and could ascertain that, although these germs are always present in the atmosphere, as results from the experiments of Pasteur, their number depends on incessant variations.

"Accordingly, *the number of atmospherical bacteria, very small in winter, increases in the spring, reaches its height during summer and the first port of autumn, and rapidly diminishes during frost;* the same law can be applied to the sporules of fungi, but, while these are numerous during damp weather, the quantity of bacteria is at the same time diminished, and rises again when the soil becomes dry; precisely during the time when the sporules of fungi become rare.

"Until we are able to prepare a liquid in which the germs of all sorts of bacteria can grow equally well, it will be difficult to know the exact number of bacteria contained in a given volume of air. On experimenting with neutral broth, totally deprived of organisms, it results that the average number of bacteria living in a cubic meter of air, does not exceed 200; this would lead us to believe that the atmosphere contains 100 times more sporules than bacteria. But unluckily, experience proves every day that the medium of nutrition has a great influence over the growth of bacteria; here is an instance, the *bacillus ureæ*, a very active agent of ammoniacal fermentation, and an organism perfectly distinct from the *micrococcus* ferment of urea, studied by Pasteur and Van Tiegen, grows well in urine, and in liquids containing urea, but is unable to grow in neutral broth. However it may be, the last liquid is altogether a favorable medium for the growth of a great number of species, and gives results whose greatest merit is a comparative one.

"During summer and autumn, times happen when a thousand bacteria are found in a cubic meter of air at Montsouris, (Fe ). In winter, this number often decreases to four or five, and days come when the dust contained in 200 liters of air is incapable of producing the infection of the liquids most easily affected.

"Inside of buildings, mechanical agency, as currents of air, being excluded, it requires from 30 to 50 liters of air to determine some change in the liquids; in my laboratory, the dust of five liters of air usually alters neutral broth; in the sewers of Paris, the infection of the same liquid is produced by the germs held in a liter of air.

"These results are very different from those published by Tyndall, according to whom a few cubic centimeters of air would in most cases be sufficient to infect various infusions. The interest brought to bear on bacteria, the presumed cause of infectious diseases, has led me to compare the number of deaths, caused by that class of diseases in Paris, with that of the bacteria contained in the atmosphere during the corresponding period. From such a comparison, extending from December, 1879, to June, 1880, it results that *every increase in the bacteria of the air is followed eight days later by an increase in the deaths from contagious and epidemic diseases.*

"I will prove later that contrary to the opinion held by many authors, watery vapor arising from the soil, the rivers, and putrefying masses, is always pure, that the gases arising from buried cadavers, are always deprived of bacteria; that the foul air directed through putrefying meat, far from loading itself with bacteria, is entirely cleaned, provided the infectious and putrid filter remains as damp as the soil, 30 cm. beneath the surface.

"Before closing, I must grant that to this day, none of the numerous species of bacteria which I isolated and inoculated into living animals has given rise to any pathological process worthy to be mentioned."

These propositions would acquire a greater importance if the experimenter had been able to prove that all species of bacteria are subjected to the same laws of existence, but that is not at all probable. Applying to them the law of natural selection, we can easily infer that the growth of one kind of bacteria excludes to a great extent that of another. This is the more probable as statistics prove that an epidemic of one or two of the infectious diseases is generally accompanied by a diminution in other diseases belonging to the same class, in the same locality.

The promises of Dr. Miguel are very brilliant, and we may still expect better results in that branch of inquiry. The truth is that this subject is not by any means exhausted, and that the germ theory, like all scientific achievements, requires a great deal of material and skill for its construction.

186 BLUE ISLAND AVE.

